# Personalized Physiological Medicine as the Future of Intensive Care Medicine

**DOI:** 10.2478/jccm-2022-0020

**Published:** 2022-08-12

**Authors:** Leonard Azamfirei

**Affiliations:** 1 Department of Anesthesiology and Intensive Care George Emil Palade University of Medicine, Pharmacy, Science, and Technology of Târgu-Mures, Târgu-Mures Romania

Personalized medicine has become increasingly popular over the last decade as a response to the variability of patients’ responses to certain medical or technological therapies [1]. The effectiveness of randomized controlled trials has become increasingly controversial. While personalized medicine came to be associated with the field of oncology, critically ill patients have also become a beneficiary of personalized medicine. Patients admitted to the intensive care unit have a series of multiple and intricate dysfunctions as well as complex and rapidly changing pathophysiological mechanisms underlying their disease. That is the background from which the concept of personalized physiological medicine (PPM) emerged. It is directly related to the therapy needs of a patient and their physiological status, determined by a certain genetic profile and characterized by biomarkers, specific to the individual and not exclusive to the disease [2, 3, 4]. Monitoring of organs becomes insufficient without continuous monitoring of microcirculation, cell metabolism, and the entire cellular functionality; the translation of these dynamic data into quantifiable parameters requires predominantly in vivo monitoring.

The cornerstones of PPM are [5]:

1. Fitness and frailty - muscle status, at the organ level or the level of myokine generating myocytes, is directly correlated with the cellular fragility from which cellular dysfunction starts and the unique response to the critical illness state is mediated.
2. Organ function response to therapy - requires a different category of markers—physiological biomarkers—which must precede pharmacological biomarkers as more early indicators of cellular dysfunction and therapeutic efficacy.
3. Hemodynamic coherence - intrinsically related to perfusion and implicitly to tissue oxygenation. Recent hand-held vital microscopes (HVM) can assess microvascular reactivity and physiological reserve [6].
4. Integration and feedback - the response at the cellular level must be captured and converted into usable signals, with the help of in vivo biosensors with ex vivo wireless communication, and then integrated into mathematical models. For biological systems that have a high rate of change, the mathematical integration of these signals is subject to the Nyquist-Shannon sampling theorem whereby the variable under evaluation must be sampled at least twice at the highest rate of change of the system, which in clinical translation means, in effect, continuous monitoring [7].

Biosensors must monitor the cellular dysfunction underlying organ failure. Structurally, they must replicate the model of common sensors, with the following components: target/analyte captured by recognition/capturing receptor, which results in a change in the transducer surface (antibodies, enzymes, peptides, DNA/LNA, RNA, etc.). The transducer (optical, electrochemical, mechanical, or magnetical) induces a recognition event that translates the capturing event into an electrical signal [8].

To create these biosensors capable not only of providing additional diagnostic elements, but also of providing drug therapy according to a new concept - theragnostic drug delivery - new generations of nanoparticles with intracellular adhesion, communication, and mobility control mechanisms are needed. Emerging technologies, such as specific carbon nanotube (CNT) materials or biomolecules derived from cells, bacteria, or viruses embedded in the electronic component of biosensors (synthetic biology), with the ability to emit signals when they reach their therapeutic target, can be used to achieve this goal [9].

Intensive care in the future will take advantage of the genetic sequencing that underpins personalized medicine and hence pharmacogenetic therapy. Imaging will have to move out of the organ paradigm and identify the multi-organ complex and interaction, based not only on the morphological aspect but especially on the functional, physiological one. The data obtained will be evaluated outside individual clinical reasoning, on machine learning models. The resulting solutions, including organ reconstruction, will be based on bioprinting, decellularized biomatrices, and 3D cell cultures with custom-purpose nanoparticles.

A 2016 JAMA editorial stated: ‘’Invest in and apply the promise of cognitive computing with rapidly expanding computing capability to integrate, process and assess very large databases, opportunities develop for accelerated learning, understanding individual variation and developing predictive modeling” [10]. For those analyzing this data, the collected variables are defined by 5 main characteristics: velocity (the frequency at which data is generated, captured, and shared), volume (big data – often containing terabytes of information), variety (unstructured images, videos, text files, and monitoring output), veracity (quality and origin of data) and value. They are analyzed using artificial intelligence algorithms.

Intensive care is a multidisciplinary field that does not belong exclusively to intensivists. The medical and technical aspects of healthcare are often siloed, with doctors only interested in the medical component and engineers only interested in the technical aspect. This can make it difficult to understand the physiological process. A multidisciplinary integration is necessary to advance personalized medicine. The future of intensive care is the migration from volume to value, to quality, i.e. back to the physiology from which all therapies originate.
